# Supplementary material for: A systematic comparison of eight new plastome sequences from Ipomoea L
Source: PeerJ. 2019 Mar 11;7:e6563. doi: 10.7717/peerj.6563 (PMC6417408; doi:10.7717/peerj.6563)
Supplement: Supplemental Information 1 [file peerj-07-6563-s001.docx]

Table S1. GenBank accession number/Plant ID for taxa used in comparative analysis and phylogenetic tree construction in this study.

| Species | GenBank accession number / Plant ID |
| --- | --- |
| Ipomoea nil | AP017304 |
| Ipomoea purpurea | EU118126 |
| Ipomoea batatas | NC_026703 |
| Ipomoea amnicola | KF242478 |
| Ipomoea argillicola | KF242479 |
| Ipomoea cairica | KF242480 |
| Ipomoea diamantinensis | KF242481 |
| Ipomoea dumetorum | KF242482 |
| Ipomoea eriocarpa | KF242483 |
| Ipomoea hederifolia | KF242484 |
| Ipomoea murucoides | KF242486 |
| Ipomoea orizabensis | KF242488 |
| Ipomoea pedicellaris | KF242489 |
| Ipomoea pes-caprae | KF242490 |
| Ipomoea polpha | KF242491 |
| Ipomoea setosa | KF242492 |
| Ipomoea ternifolia | KF242494 |
| Ipomoea tricolor | KF242495 |
| Ipomoea minutiflora | KF242498 |
| Ipomoea obscura | KF242499 |
| Ipomoea pes-tigridis | KF242500 |
| Merremia quinquefolia | KF242501 |
| Operculina macrocarpa | KF242502 |
| Ipomoea alba | JRIW_27755* |
| Ipomoea biflora | AH 302* |
| Ipomoea gracilis | ME 2940* |
| Ipomoea grandifolia | CIP460322* |
| Ipomoea indica | BH 19551* |
| Ipomoea malvaeoides | EH 6116* |
| Ipomoea littoralis | Anon 152892* |
| Ipomoea ramosissima | JRIW 27942* |
| Ipomoea sepacuitensis | MP 1020* |
| Ipomoea tenuissima | AHC 495* |
| Ipomoea tiliacea | CIP460524* |

* indicate the species from Muñoz-Rodríguez et al., (2018, Current Biology)
