# Supplementary figures and images for: A systematic comparison of eight new plastome sequences from Ipomoea L
Source: PeerJ. 2019 Mar 11;7:e6563. doi: 10.7717/peerj.6563 (PMC6417408; doi:10.7717/peerj.6563)

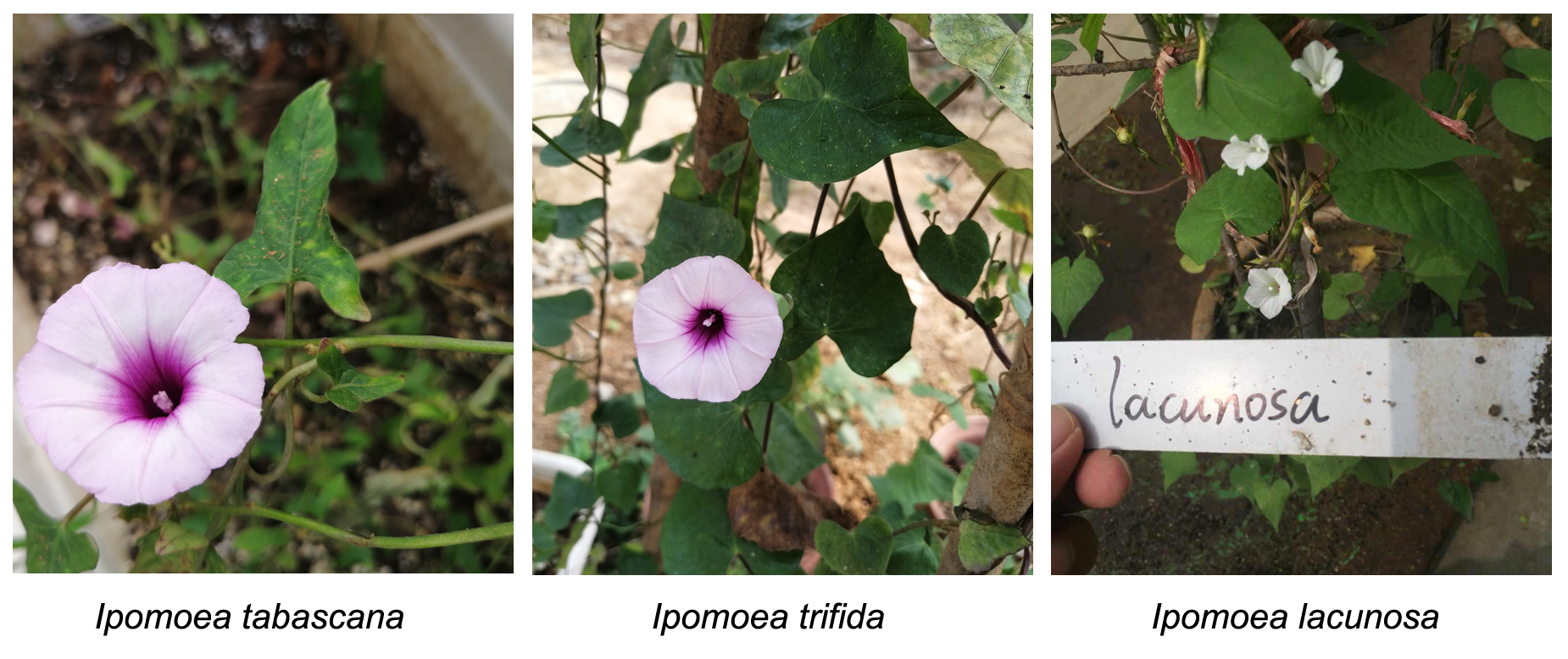

Supplement: Supplemental Information 8 [file peerj-07-6563-s008.jpg]
